# Multimodality Imaging Assessment of Desmoid Tumors: The Great Mime in the Era of Multidisciplinary Teams

**DOI:** 10.3390/jpm12071153

**Published:** 2022-07-16

**Authors:** Igino Simonetti, Federico Bruno, Roberta Fusco, Carmen Cutolo, Sergio Venanzio Setola, Renato Patrone, Carlo Masciocchi, Pierpaolo Palumbo, Francesco Arrigoni, Carmine Picone, Andrea Belli, Roberta Grassi, Francesca Grassi, Antonio Barile, Francesco Izzo, Antonella Petrillo, Vincenza Granata

**Affiliations:** 1 Division of Radiology, Istituto Nazionale Tumori IRCCS Fondazione Pascale—IRCCS di Napoli, 80131 Naples, Italy; igino.simonetti@istitutotumori.na.it (I.S.); s.setola@istitutotumori.na.it (S.V.S.); c.picone@istitutotumori.na.it (C.P.); a.petrillo@istitutotumori.na.it (A.P.); 2 Department of Applied Clinical Sciences and Biotechnology, University of L’Aquila, 67100 L’Aquila, Italy; federico.bruno.1988@gmail.com (F.B.); carlo.masciocchi@univaq.it (C.M.); antonio.barile@univaq.it (A.B.); 3 Italian Society of Medical and Interventional Radiology (SIRM), SIRM Foundation, 20122 Milan, Italy; palumbopierpaolo89@gmail.com; 4 Medical Oncology Division, Igea SpA, 80013 Napoli, Italy; r.fusco@igeamedical.com; 5 Department of Medicine, Surgery and Dentistry, University of Salerno, 84084 Salerno, Italy; carmencutolo@hotmail.it; 6 Hepatobiliary Surgical Oncology Division, Istituto Nazionale Tumori IRCCS Fondazione Pascale—IRCCS di Napoli, Via Mariano Semmola, 80131 Naples, Italy; dott.patrone@gmail.com (R.P.); a.belli@istitutotumori.na.it (A.B.); f.izzo@istitutotumori.na.it (F.I.); 7 Department of Diagnostic Imaging, Area of Cardiovascular and Interventional Imaging, Abruzzo Health Unit 1, 67100 L’Aquila, Italy; 8 Emergency and Interventional Radiology, San Salvatore Hospital, 67100 L’Aquila, Italy; arrigoni.francesco@gmail.com; 9 Division of Radiology, Università degli Studi della Campania Luigi Vanvitelli, 80127 Naples, Italy; roberta.grassi@policliniconapoli.it (R.G.); francesca.grassi1@studenti.unicampania.it (F.G.)

**Keywords:** desmoid tumors, aggressive fibromatosis, magnetic resonance imaging, ultrasound, computed tomography, diffusion-weighted imaging, dynamic contrast enhanced-MRI

## Abstract

Desmoid tumors (DTs), also known as desmoid fibromatosis or aggressive fibromatosis, are rare, locally invasive, non-metastatic soft tissue tumors. Although histological results represent the gold standard diagnosis, imaging represents the fundamental tool for the diagnosis of these tumors. Although histological analysis represents the gold standard for diagnosis, imaging represents the fundamental tool for the diagnosis of these tumors. DTs represent a challenge for the radiologist, being able to mimic different pathological conditions. A proper diagnosis is required to establish an adequate therapeutic approach. Multimodality imaging, including ultrasound (US), computed tomography (CT) and Magnetic Resonance Imaging (MRI), should be preferred. Different imaging techniques can also guide minimally invasive treatments and monitor their effectiveness. The purpose of this review is to describe the state-of-the-art multidisciplinary imaging of DTs; and its role in patient management.

## 1. Introduction

Desmoid tumors (DTs), also known as desmoid fibromatosis or aggressive fibromatosis, is an unusual and locally aggressive monoclonal, fibroblastic proliferation characterized by a variable and often unpredictable clinical course. According to the World Health Organization (WHO), DT is a “clonal fibroblastic proliferation that arises in the deep soft tissues and is characterized by infiltrative growth and a tendency toward local recurrence but an inability to metastasize”, even though it may be multifocal in the same limb or body part [1]. Although histopathological analysis represents the gold standard for diagnosis [1,2,3], imaging represents a necessary tool during the multidisciplinary approach to these tumors since it allows, thanks to the possibility of multimodality assessment (ultrasound (US), computed tomography (CT) and Magnetic Resonance Imaging (MRI)) [4,5,6,7,8,9,10], the detection, localization and evaluation of adjacent structures involvement, to establish a differential diagnosis so as to guide management (surgical or minimal invasive) [11,12,13,14,15,16,17,18,19,20,21,22,23,24,25].

The purpose of this review is to describe state-of-the-art nature of multimodality imaging evaluation, highlighting the advantages and disadvantages of the different techniques in abdominal and extra-abdominal DTs. We assessed their role during the different phases of patient management, including the possibility of guiding interventional radiology treatment.

## 2. Epidemiology and Histopathology

Desmoid tumors commonly affect individuals between the age of 15 and 60 years, with a peak incidence at 35–40 years and a reported incidence of 2–4 per million population, mainly in women of reproductive age [1,2,3]. The etiopathogenesis of DTs is unclear, although it is believed to be multifactorial [1,2,3]. DT may be sporadic or familial. Trauma, pregnancy and the use of oral contraceptives have been implicated in etiopathogenesis [1,2,3]. Although pregnancy and the use of oral contraceptives have been shown to be associated with the development of DT, the exact role of hormonal influence is not fully understood [1,2,3]. These lesions can involve different types of connective tissues, including muscle, fascia and aponeurosis. The most common sites are the abdominal wall, abdominal mesentery limbs and girdles [1,2,3,26,27,28,29,30,31,32,33]. These lesions can infiltrate the surrounding tissues and organs, spreading across the various floors muscle structures and although they have a low tendency to metastasize, they have a high propensity for local recurrence. Therefore, this tumor has now been classified as an “intermediate, locally aggressive” tumor in the WHO classification of soft tissue tumors [1,2,4].

To date, two different clinical–pathological entities have been identified: sporadic DT and DT associated with adenomatous polyposis coli (APC) gene mutation [1]. Most of these cancers arise as sporadic variants. Sporadic tumors are more frequently extra-abdominal [2,34,35,36,37]. Several studies show that the inherited variant can be seen in 5–16% of patients with familial adenomatosis polyposis (FAP) [1,2,3]. FAP-related DTs lesions are mainly in the abdominal cavity (about 80%), abdominal wall (10–15%) and extra-abdominal (about 5%). [1,2,3] Intra-abdominal DTs are mostly located in the mesentery. These tumors grow slowly, generally increasing by 2 to 9 cm per year, and locally without metastasizing distantly. Despite their benign nature, they can be infiltrative and multifocal, causing significant morbidity and mortality [1,2]. In addition, DT associated with FAP tends to have multifocal lesions, larger and most commonly occurs in younger patients [1,2,3].

It was demonstrated that a third variant known as “wild-type” DT (without CTNNB1 or APC mutations) does not exist and is the result of a diagnostic error (other proliferations of spindle cells that mimic DT) or DT with unrecognized CTNNB1 or APC mutations [1,2,3].

The definitive diagnosis is histopathological with evidence of the proliferation of uniform spindle cells resembling myofibroblasts in the background of abundant collagenous stroma and the vascular network and characterized immunohistochemistry stains positive for nuclear B-catenin, vimentin, cyclooxygenase 2, tyrosine kinase PDGFRb, androgen receptor and estrogen receptor beta but negative for desmin, S-100, h-caldesmon, CD34 and c-KIT [1,2].

## 3. Clinical Presentation and Treatment

The clinical presentation of DTs is variable and correlates with tumor location [1,2,3].

**Figure 1:**
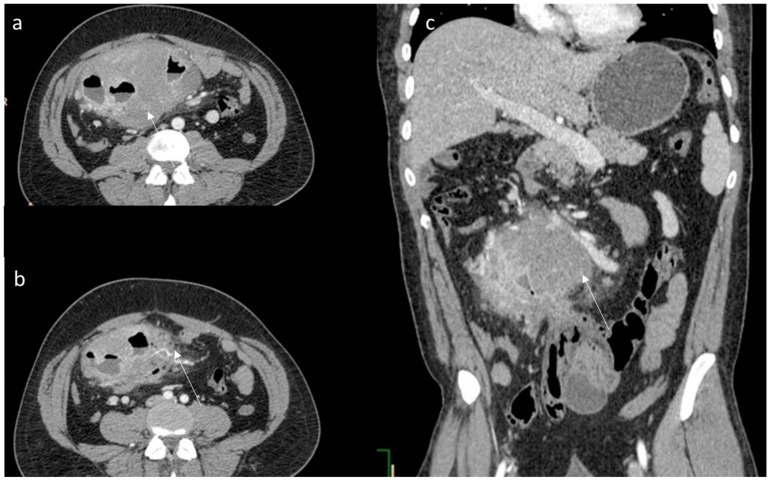
(**a**,**b**) CT assessment (arrow) of aggressive fibromatosis; the arrow shows lesion in axial; (**c**) MPR coronal plane of portal phase of contrast study. The lesion enhancement is mild to moderate with involvement of intestinal loops.

Generally, DTs have a chronic progression, remaining asymptomatic for a long time and developing into a solid lesion which might present with pain. However, larger lesions and those adjacent to neurovascular structures may be associated with pain and functional impairment [1,2,3]. Desmoid tumor complications correlate to their locally aggressive character, causing compression and/or invasion of the adjacent organs and tissues (Figure 1). Intra-abdominal DTs may determine bleeding, intestinal obstruction, perforation and, infrequently, an abscess [1,2,3].

Most DTs in the abdominal wall and extra-abdominal sites may present as a painless mass. Extra-abdominal DTs (EADTs) localization is more common in the head and neck region, where involvement of the airways or major vessels can present with hoarseness, dyspnea or, in extremely rare cases, be fatal [37,38,39,40,41]. Follow localizations at limbs, presenting as palpable masses, severe pain or muscle contractures and at the thoracic and abdominal wall [42,43,44,45,46,47,48,49,50].

Surgical resection may be more complicated for intra-abdominal DTs compared to extra-abdominal and abdominal wall lesions. In fact, resection may be technically challenging, particularly in patients with FAP. So, surgery has lost its traditional role as a first-line treatment of the disease, and several other treatment methods are being considered [51,52,53,54,55,56,57,58,59,60,61]. In fact, for asymptomatic patients, close observation by serial imaging should be initiated with an interval of 3 to 6 months, given the variable nature of DTs, including the possibility of spontaneous regression. Despite this, primary surgery with negative margins was considered, in the past, the standard of care. However, due to the pattern of infiltrative growth, the scope of resection needed to achieve negative margins could often lead to important function impairments and cosmetic alterations, which are not acceptable in an indolent disease. Furthermore, the efficacy of marginal R1 resections remains unclear. A positive surgical margin was found to be an adverse predictor of worse local control in some series but not in others [3,34]. Several researchers showed that progression-free survival curves were not significantly different based on the microscopic assessment of surgical resection quality (R0 versus R1), although R2 resections resulted in a significantly poorer prognosis [3,34]. Other prognostic factors associated with poor PFS were age younger than 37 years, tumor size larger than 7 cm and extra-abdominal localization, especially tumors found in the distal extremities [3,34]. Based on these data, French [60] and Italian sarcoma groups [11] did not recommend surgery as upfront therapy, except in the case of the patient’s preference. So, increasing attention has been directed toward initial non-operative management, including watchful waiting using nonsteroidal anti-inflammatory drugs (NSAIDs) with or without hormonal manipulation, chemotherapy or radiation therapy [3,11,34,60].

With regard to radiation therapy, this approach should be considered for tumors located at critical sites (such as the head and neck, limb girdles and pelvis), for which surgery would involve functional impairment, or for inoperable, symptomatic/progressive disease that did not respond to other therapeutic approaches, radiotherapy alone could be preferable over other local treatments [3]. Adjuvant radiotherapy is recommended for extremity/limb girdle disease after R1/R2 resection for recurrent disease or following surgery at critical sites (i.e., head and neck), regardless of margins status.

Aggressive chemotherapy should be avoided because it is associated with significant morbidities. However, cytotoxic chemotherapy, non-cytotoxic systemic therapy and targeted therapy have been revealed as part of different treatment regimens [62,63,64,65,66,67,68,69,70,71,72]. Cytotoxic chemotherapy is usually the first treatment option for rapidly growing and symptomatic unresectable or advanced diseases. The most frequently used regimens include methotrexate and vinblastine in combination and an anthracycline-based regimen [3]. Recent progress regarding DT biology and molecular pathways has led to the development of promising novel biological agents. In any case, a multidisciplinary approach is required and is gradually employed, especially in intra-abdominal DTs [62,63,64,65,66,67,68,69,70,71,72]. In addition, recent studies in the literature have shown that EADTs, following a correct surgical excision of the lesion with undamaged surgical margins, have a low rate of local recurrence and distant metastasis. The relationship between age, sex and local recurrence prognosis is controversial. Conversely, tumor size can be considered a possible risk factor for a poor prognosis, as tumors > 5 cm in size have a higher recurrence rate [2,3,4]. Occasionally, surgical management is the only option in complicated patients [73,74,75,76,77,78,79].

## 4. Imaging

Since the management of DTs mandates a multidisciplinary approach, imaging plays a pivotal role in the detection and assessment of these lesions. In the correct radiological disease management, multimodality imaging, including ultrasound (US), Computed Tomography (CT) and Magnetic Resonance Imaging (MRI), should also be preferred concerning the different phases of DTs approaches [80,81,82,83,84,85,86,87,88,89,90,91,92]. In fact, during radiologist work-up, different moments may be considered: detection and characterization, adjacent structures involvement assessment, treatment response evaluation and surveillance [93,94,95,96,97,98,99,100,101,102,103,104,105,106,107,108,109,110]. During each of these moments, the different techniques can be associated with and/or follow each other.

## 5. Ultrasound Assessment

Ultrasound (US) is an inexpensive tool, widely available and safe since it does not use ionizing radiation so that the examination can be repeated several times, even in risk categories such as children and pregnant women [111,112,113,114,115,116]. US plays a limited role mainly in the delineation of mass and lesions involving the abdominal wall, chest wall, breast and extremities. However, due to operator dependence and the poor performance of small intra-abdominal lesions, the necessity for patient collaboration reduces the sensitivity and specificity both in detection and characterization [111,112,113,114,115,116].

On US assessment (Table 1), these lesions show a variable appearance ranging from well-circumscribed to poorly defined infiltrative heterogeneous solid mass with variable echogenicity depending upon the amount of collagen, fibrosis and cellular components within the lesion. Vascularity is variable, as manifested at Color Doppler US or Contrast-enhanced US (CEUS) [80]. DT may be associated with a fascial tail sign, indicating thin linear extension along fascial planes and the staghorn sign from intramuscular fingerlike extensions of the tumor [80]. Sometimes DTs appear as irregular, speculated, hypoechoic masses with Color Doppler flow mimicking malignancy [117,118,119]. US can be used to guide ablation treatment in DT unfit for surgical resection [120]. Radiofrequency ablation (RFA) is the most frequently employed ablation tool, and its success is essentially due to the minimally invasive nature of the treatment with lower complication rates, superior toxicity profiles and often comparable or superior mid- and long-term oncologic outcomes compared to conventional therapies such as surgical procedures or systemic treatments [121,122,123,124,125]. There are few reports on the RFA treatment of DTs with relatively small volumes in superficial tissues, such as the abdominal wall, limbs and trunk [126,127,128,129]. In these cases, US is a promising tool for planning, targeting, monitoring, intra-procedural modification and assessing treatment response, including technical success, treatment efficacy and complications [130,131,132,133,134].

To the best of our knowledge, one study described CEUS appearance in abdominal DT [135], with the early enhancement of the contrast agent and very long wash-out, a typical pattern of benign lesions probably due to the presence of fibrotic tissue [135]. Xu et al. described CEUS appearance in 19 cases of superficial DF: the tumors were hyperenhanced, with an enhanced pattern of rapid wash-in and slow wash-out [136].

**Table 1:** Imaging features of abdominal and extra-abdominal DTs and advantages and weaknesses of diagnostic tools.

Desmoid Tumor	US	CT	MRI
Abdominal features [80]	Variable appearance ranging from well-circumscribed to poorly defined infiltrative heterogeneous solid mass with variable echogenicity. Vascularity is variable. At CEUS, early enhancement of the contrast agent and a very long washout	CT findings of intra-abdominal lesions are determined by the amount of collagen and myxoid tissue; therefore, the myxoid component of the tumor tends to be hypodense compared to skeletal muscle, while the collagen and fibrotic component may be isodense or hyperdense. After intravenous contrast administration, the enhancement is mild to moderate	Heterogeneous pattern, with signal iso- to hyperintense to skeletal muscle on T2-weighted images and isointense to muscle on T1-weighted images. Decreased signal intensity on T2-weighted images most likely results from dense collagen and hypocellularity; conversely, increased T2 signal intensity reflects a high content of spindle cells. DTs commonly (90%) show moderate to intense contrast enhancement, especially in the more cellular and less fibrotic regions.
Extra-abdominal Features [137]	Variable appearance from well-circumscribed to poorly defined infiltrative heterogeneous solid mass with variable echogenicity. Vascularity is variable. At CEUS, early enhancement of the contrast agent and a very long washout	Slightly lower density, a higher degree of enhancement and unclear boundaries	Extra-abdominal DTs typically occur in the intermuscular location along deep fascia and may show a thin rim of surrounding fat (split fat sign), linear enhancing extension along the fascial planes, and feathery margins resembling a flame (flame sign).
Advantages	Inexpensive; widely available; safe [111,112,113,114,115,116]	Requires high spatial resolution to obtain sufficient anatomical detail for the detection of deep lesions and for targeting interventional procedures [80]	Multiparametric approach; the exceptional contrast resolution; functional assessment [138,139,140,141,142,143]
Weakness	Operator dependence; poor performance for small intra-abdominal lesions; patient’s collaboration [111,112,113,114,115,116]	Radiation exposure [97]	Long examination and interpretation time; high costs [97]

## 6. Computed Tomography Assessment

CT has a dual approach: diagnostic and therapeutic. The sensitive advantage of the use of CT is to have a high spatial resolution and obtain sufficient anatomical detail for the detection of deep lesions and for targeting interventional procedures [144,145,146]. A recent technique, dual-energy CT (DECT), was established to increase tumor detection [147,148]. DECT, which is founded on the instantaneous acquisition of two image datasets at different energy levels, can produce virtual monochromatic images (VMIs) [149]. Additionally, thanks to DECT, radiation and contrast media doses are lower compared to conventional CT, which is mainly beneficial for patient surveillance [149].

CT is commonly used to image DTs (Table 1 and Table 2), particularly for intra-abdominal localizations for diagnosis and follow-up, as well as in preoperative assessment to identify the relationship of the tumor with adjacent neurovascular structures and organs. CT can provide critical information required for treatment planning. In addition, complications such as bowel obstruction, bowel ischemia and hydronephrosis are readily identified on CT. However, CT contributes up to 65% of medically induced radiation exposure, and this is a main critical point that should be considered during follow-up in young patients [97]. In addition, the administration of intravenous contrast media (CM) is an integral element of many CT examination protocols [97]. However, CM administration is also accompanied by a potential risk for adverse reactions, in particular, allergic reactions and contrast-induced nephropathy. Therefore, CM administration should be scrutinized, and the lowest adequate dose should be used [97].

**Figure 2:**
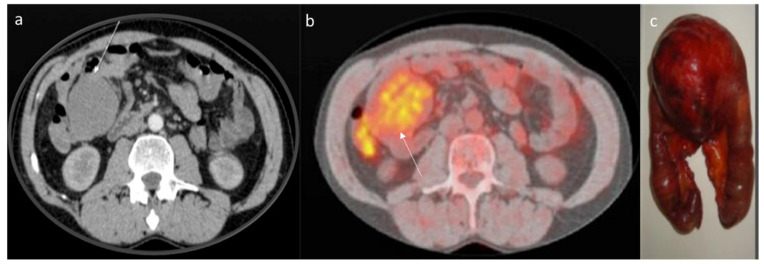
CT assessment (arrow) of mesenteric DT (**a**) in portal phase of contrast study. The lesion enhancement is mild to moderate. The 18-FDG (**b**) assessment (arrow) with moderate uptake. Surgical sample (**c**).

The CT findings of intra-abdominal lesions are determined by the amount of collagen and myxoid tissue; therefore, the myxoid component of the tumor tends to be hypodense compared to skeletal muscle, while the collagen and fibrotic component may be isodense or hyperdense. After intravenous contrast administration, enhancement is mild to moderate (Figure 2) [80]. Necrosis and calcifications are extremely rare.

**Figure 3:**
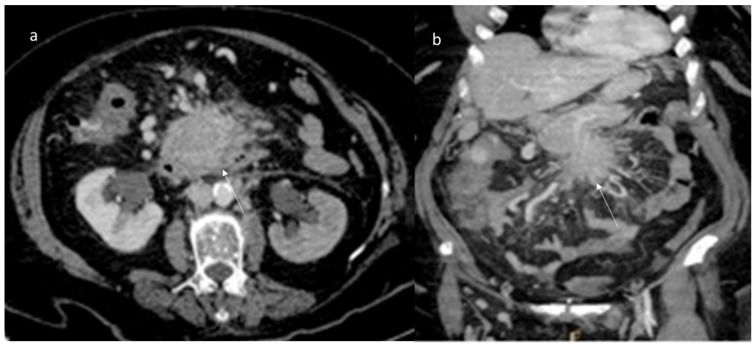
Axial (**a**) and MPR coronal (**b**) CT assessment of aggressive fibromatosis (arrow) in portal phase of contrast study. The lesion shows mild enhancement and involvement of blood vessels.

Shi et al. evaluated the imaging features in 13 patients with desmoid fibroma of the extremities, finding that the tumors showed a lower density (69.23%), a higher degree of enhancement (61.54%) and unclear boundaries (84.62%) (Figure 3); a CT value < 50 Hu was encountered in 53.85% of lesions, and the enhancement was uneven in 53.85% of cases [137].

To the best of our knowledge, no one study described the role of DECT in DTs.

As in US, CT also plays a pivotal role in guiding ablative treatment. In particular, CT-guided cryoablation is safe, effective and offers some important advantages. First, it presents the possibility of treating even large and multiple lesions in one session, creating a large area of ablation with low risks of spreading the pathological cells. Second, the technique is minimally invasive and requires low hospitalization times and mild anesthesia. Third, the functional recovery is satisfyingly immediate. Fourth, the procedure can be repeated with no risks for the patient in case of partial treatment [150,151,152].

## 7. Magnetic Resonance Imaging Assessment

**Figure 4:**
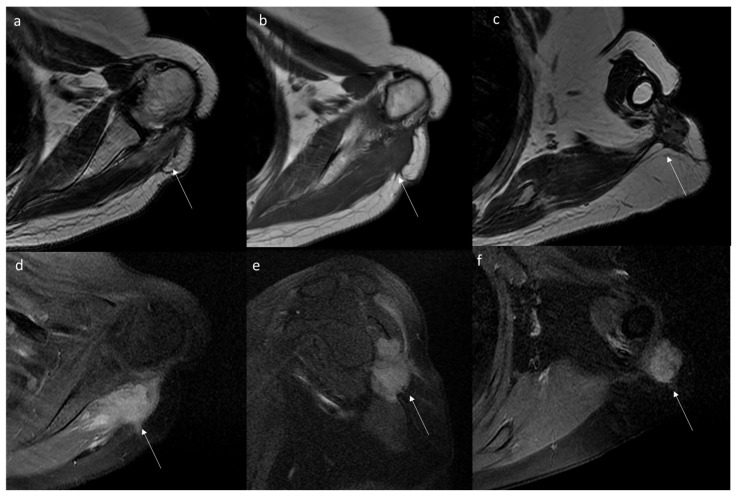
Aggressive fibromatosis of the shoulder within the muscular and fascial planes of the supraspinatus and deltoid muscles depicted (arrows) on axial T2 (**a**) and T1 (**b**,**c**) sequences and after gadolinium (**d**–**f**).

**Figure 5:**
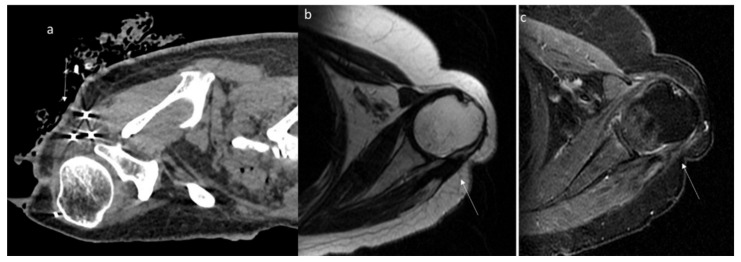
The same patient of Figure 4, treated by CT-guided cryoablation (probes and ice-ball within the lesion in (**a**); the follow-up control after 6 months depicts evident volumetric and enhancement reduction (arrows) of the lesion (**b**,**c**).

Thanks to the multiparametric approach, the exceptional contrast resolution and the possibility to exploit several advanced sequences, magnetic resonance imaging is the diagnostic gold standard for the study, characterization and follow-up of extra-abdominal DTs, with a pivotal role for intra-abdominal ones [138,139,140,141,142,143]. Most advantages are evident in particular extra-abdominal lesions occurring in the extremities, head and neck, abdominal and chest wall and in lesions at mesenteric localization in patients allergic to contrast agents or in young patients to reduce radiation exposure [1,7,21]. The signal intensity of MRI reflects the proportion of collagen fibers, spindle cells and extracellular matrix present and varies with imaging sequences. The commonly observed MR imaging appearance is a heterogeneous pattern, with signal iso- to hyperintense in the skeletal muscle on T2-weighted images and isointense in the muscle on T1-weighted images. Decreased signal intensity on T2-weighted images most likely results from dense collagen and hypocellularity; conversely, increased T2 signal intensity reflects a high content of spindle cells. DTs commonly (90%) show moderate to intense contrast enhancement (Figure 4 and Figure 5), especially in the more cellular and less fibrotic regions; however, areas of non-contrast enhancement related to necrosis may rarely be present (Table 1 and Table 2) [80].

**Figure 6:**
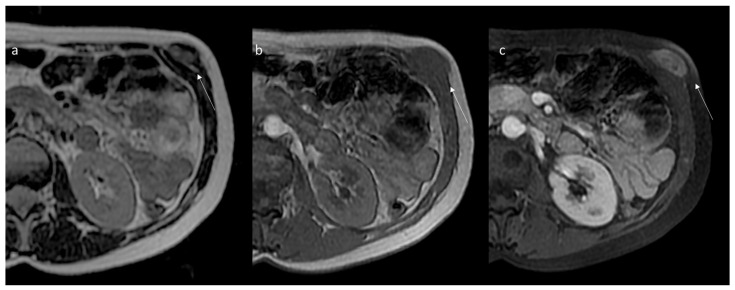
Desmoid of the anterior abdominal wall. MRI axial slices in T2 (**a**), T1 (**b**), and gadolinium-enhanced T1 (**c**) sequences. A nodular lesion is evident within the muscular and fascial planes of the internal and external oblique muscles (arrow).

Some characteristic but not specific findings of DTs on MRI have been identified. Low-signal-intensity non-enhancing linear bands in all sequences, known as the band sign, are present in 60% to 90% of DTs and can be seen in other benign (giant cell tumor of the tendon sheath) and malignant (myxofibrosarcoma and malignant fibrous histiocytoma), likely corresponding to the dense collagenous stroma often found at histologic examination [80]. Extra-abdominal DTs typically occur in the intermuscular location along deep fascia (Figure 6) and may show a thin rim of surrounding fat (split fat sign) (83% of DTs), linear enhancing extension along the fascial planes and feathery margins resembling a flame (flame sign) [80].

Beyond information on morphology, several MRI sequences can be used to obtain functional, ultrastructural information on tissue and deepen the diagnosis.

Using DWI imaging, the DWI signal and the ADC values reflect the cellularity of the tissues, so even if there are no normal cutoff values, DWI can characterize the biological activity of the tissues [153,154,155,156,157,158,159,160]. In the study of musculoskeletal soft tissue tumors, this means that, as a general rule, benign tumors with a low degree of biological activity will have a loss of ADC signal as the b values increase, while malignant tumors (in which the water has greater restriction in movement) will show high intensity at high b values [161,162]. The mean ADC of DTs was found to be significantly higher than that of malignant soft tissue tumors without overlap in the minimum ADC values [163]. DWI has also proved very useful in the assessment of treatment response [66,96]. Similarly, the evaluation of enhancement patterns can be challenging, as both granulation and scar tissues (aspecific tissue changes after chemo/radiotherapy) are enhanced after contrast administration, and the differentiation from the viable tumor is not always direct. DWI was demonstrated to improve this discrimination earlier than conventional imaging, as solid tumors are characterized by high cellularity with intact cell membranes, while tissues after cytotoxic treatment show lower cellularity and membrane damage. DWI also implements standard morphological sequences in the evaluation of postsurgical follow-up, aiding in detecting residual/recurrent tumor tissue [42,94].

Dynamic perfusion MRI is another functional imaging technique often used to evaluate tumors, mainly to depict the early intravascular and interstitial distribution of gadolinium [164,165,166,167,168,169,170,171,172]. In DTs, despite considerable variability, a time-intensity curve characterized by rapid early enhancement followed by a plateau was described [80]. However, despite the characteristic imaging results of DTs on MR imaging, a biopsy is required for histological characterization. In addition, the histological characteristics of DTs can vary over time and are reflected in MR imaging and are useful as a support in evaluating response to treatment [80].

MRI can guide ablative treatment as US and CT. High-intensity focused ultrasound (HIFU) ablation is a noninvasive treatment that has been successfully used for the treatment of various solid tumors [17]. In the past decade, several studies have been reported, suggesting the safety and efficacy of HIFU ablation for the treatment of DTs [173,174,175,176,177,178,179]. HIFU uses nonionizing radiation ultrasound as the physical therapy factor. Therefore, it not only has the potential of being a repeatable treatment but also has the potential to safely ablate more tumor tissue. Although a few studies have reported the safety and efficacy of HIFU in DTs, the sample size was too small. In addition, DTs can occur in any part of the body, including the extra-abdominal, abdominal wall and intra-abdominal types. The safety and efficacy of HIFU ablation for different types of DTs also should be assessed [180].

The critical weakness of MR assessment is related to the long examination and interpretation time, as well as higher costs, which still represent barriers to MRI use [97]. Abbreviated MRI protocols have emerged as an alternative to standard MRI protocols. These abbreviated protocols seek to reduce longer MRI protocols by eliminating unnecessary or redundant sequences that negatively affect the cost, examination time, patient comfort and image interpretation time [97].

## 8. Differential Diagnoses

**Figure 7:**
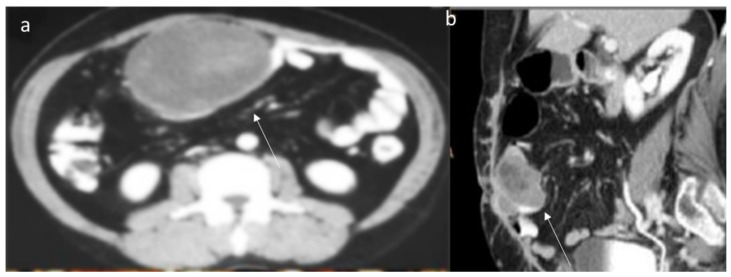
Ileal Gist (arrow) in CT portal phase of contrast study ((**a**) axial plane; (**b**) MPR sagittal plane). The lesion shows moderate enhancement with central necrosis.

**Figure 8:**
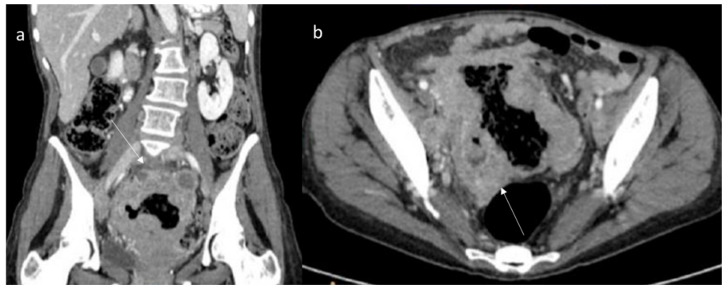
MPR coronal (**a**) and axial CT (**b**) assessment (portal phase of contrast study) of Sigmoid Lymphoma (arrow). The sigma has thickened walls with inhomogeneous contrast enhancement.

**Figure 9:**
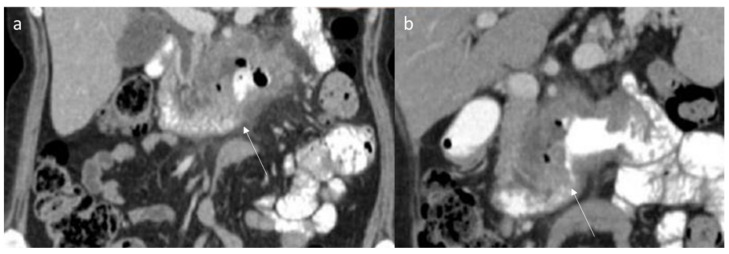
Duodenal carcinoma (arrow) in MPR coronal plane (**a**,**b**) of CT assessment during portal phase of contrast study. The duodenum has thickened walls with inhomogeneous contrast enhancement.

Regarding the abdominal wall, several pathological processes can cause wall lesions to comprise infection, hematoma, endometriosis and neoplasm [181,182]. Regarding mesenteric DT, these entities typically occur in FAP patients. However, in FAP patients with colorectal cancer, it is possible to find mesenteric metastases that mimic DT. In addition, other pathological entities should be considered in the differential diagnoses, including gastrointestinal stromal tumor (Figure 7), lymphoma (Figure 8), neuroendocrine tumor, carcinoma (Figure 9) and retractile sclerosing mesenteritis [2]. In this context, clinical history and imaging features could be helpful in lesion characterization.

Desmoid tumors in FAP patients are characterized considering clinical data, surgical history and imaging studies. CT and MRI allow us to characterize the lesion and to determine the relationship between lesions and surrounding organs for proper treatment planning. During CT study, the lesion density is uniform with uniform enhancement during the contrast study [80,137]. These features are not typically for gastrointestinal stromal tumors, lymphoma, neuroendocrine tumors and/or carcinoma, which show inhomogeneous density due to necrosis or for hormonal mesenteric reaction [80,137]. In addition, DTs have several typical features on MRI evaluation, such as a star shape and extension into the fascial planes and fat tissue in a sunburst-like form, with homogeneous signal isointense in T1-W and hyperintense in T2-W [80].

Regarding extra-abdominal DTs, several soft tissue lesions (melanoma metastases, primitive soft sarcoma, etc.), occurring in extremities, head and neck and trunk, may mimic these entities. During CT studies, DTs of the extremities show a low density, mild enhancement and unclear boundary [137]. In MRI studies, typical features are a round or fusiform shape, unclear boundaries, uniform signals, uneven enhancement, “tree root” or “claw” infiltration and invasion of the neurovascular bundles [137]. Conversely to them, malignant soft tissue tumors show inhomogeneous T1-W and T2-W signals, and the T2 signal intensity is higher than fat signal, with calcifications or cystic necrosis [183,184]. Therefore, CT and MRI studies allow us to identify typical extremities’ DTs features, although MRI provides an objective basis for the diagnosis. Additionally, MRI has a higher soft tissue contrast with clear advantages in the soft tissue tumors assessment (especially in the extremities or head and neck). It is also suitable for younger patients in whom the use of ionizing radiation should be avoided or who are allergic to iodine contrast agents. However, CT and MRI can be combined to optimize the diagnostic accuracy, as well as to reduce the incidence of missed diagnosis or misdiagnosis [183].

So, CT or MRI scans can not only help for diagnosis but also in determining the relationship between tumors and the surrounding organs to obtain proper pre-treatment planning. Otherwise, although US assessment is safe since it does not use ionizing radiation so that the examination can be repeated several times [111,112,113,114,115,116], it plays a limited role in the delineation of mass and lesions involving the abdominal wall, chest wall, breast and extremities. However, due to operator dependence and the poor performance of small intra-abdominal lesions, there is a necessity for patient collaboration to reduce the sensitivity and specificity both in the detection and characterization of these tumors.

Although imaging assessment could help characterization, a definitive diagnosis requires histopathological confirmation [2]. Pathology is the gold standard for the diagnosis of DTs. Histological examination reveals paucicellular proliferation of fibroblasts and myofibroblasts in a dense collagenous background, spindle cells with small and regular nuclei, pale eosinophilic cytoplasm and acellular central areas with increasing cellularity towards the periphery. Immunohistochemistry shows the cells are b-catenin, vimentin, Ki-67, SMA, CD68 and CD34 positive, which can assist with the diagnosis [2].

## 9. Conclusions

Desmoid tumors represent a challenge for the radiologist, being able to mimic different pathological conditions. A proper diagnosis is required to establish the proper therapeutic approach in relation to the location, clinic and evolution of the disease. Imaging plays a pivotal role in the detection and assessment of these lesions. In the correct radiological disease management, multimodality imaging, including US, CT and MRI, should be preferred. Furthermore, in relation to the different phases of DTs approaches, detection and characterization, adjacent structures involvement assessment, treatment response evaluation and surveillance should also be considered. These different imaging techniques can also guide minimally invasive treatments and monitor their effectiveness.

Regarding differential diagnoses, although imaging assessment could aid characterization, a definitive diagnosis requires histopathological confirmation.

## Figures and Tables

**Table 2:** Imaging features of abdominal and extra-abdominal DTs compared to other malignancies.

Tumor	Desmoid Abdominal Tumor	Other Abdominal Malignancy	Desmoid Extra-Abdominal Tumor	Malignant Soft Tissue Tumors
Imaging Assessment [80,137]	The density of the lesions on CT imaging is uniform, and an enhanced scan can show uniform enhancement. Homogeneous signal is isointense in T1-W and hyperintense in T2-W	Inhomogeneous density on CT and signal intensity on MRI, due to necrosis and calcifications, with inhomogeneous contrast enhancement during contrast studies	CT features of desmoid tumors of the extremities exhibited a slightly low density, mild enhancement, unclear boundary and uneven enhancement after contrast administration. Their imaging features on MRI were a round or fusiform shape, unclear boundaries, uniform signal, uneven enhancement, “tree root” or “claw” infiltration and invasion of the neurovascular bundles	Inhomogeneous density on CT and long T1 and long T2 signals, T2 signal intensity higher than that of fat on MRI. Calcification or cystic necrosis

## Data Availability

Data are available at https://zenodo.org/record/6805439#.YsZ_t4RBy3A (accessed on 13 June 2022).
